# miR-144 and targets, c-fos and cyclooxygenase-2 (COX2), modulate synthesis of PGE2 in the amnion during pregnancy and labor

**DOI:** 10.1038/srep27914

**Published:** 2016-06-14

**Authors:** Huanan Li, Jiawei Zhou, Xiajie Wei, Ran Chen, Junnan Geng, Rong Zheng, Jin Chai, Fenge Li, Siwen Jiang

**Affiliations:** 1 Key Laboratory of Swine Genetics and Breeding of Agricultural Ministry and Key Laboratory of Agricultural Animal Genetics, Breeding and Reproduction of Ministry of Education, College of Animal Science and Technology, Huazhong Agricultural University, Wuhan 430070, People’s Republic of China; 2 The Cooperative Innovation Center for Sustainable Pig Production, Wuhan 430070, China People’s Republic of China

## Abstract

Labor is initiated as a result of hormonal changes that are induced by the activation of the inflammatory response and a series of biochemical events. The amnion, which is the primary source of prostaglandin E2 (PGE2), plays an important role in the process of labor. In the present study, we uncovered a pathway in which c-fos, cyclooxygenase-2 (COX2) and miR-144 function as hormonal modulators in the amnions of pregnant mice and humans. miR-144 down-regulated the synthesis of PGE2 during pregnancy by directly and indirectly inhibiting COX2 expression and by directly inhibiting the expression of c-fos, a transcriptional activator of COX2 and miR-144. Estrogen (E_2_) activated c-fos, thus promoting the expression of miR-144 and COX2 during labor. However, the increase in COX2 resulted in the partial inhibition of COX2 expression by miR-144, thereby slightly reducing the secretion of PGE2. These observations suggest that miR-144 inhibits PGE2 secretion by section to prevent the initiation of premature labor. Up-regulated expression of miR-144, c-fos and COX2 was also observed both in preterm mice and in mice undergoing normal labor. In summary, miR-144, c-fos and COX2 play important roles in regulating PGE2 secretion in the amnion during pregnancy and labor.

Labor is an extremely complex whose mechanism has not been fully elucidated. Most, if not all, of the events that induce labor can be attributed to the action of E_2_, and evidence suggests that maternal E_2_ plays a role in triggering parturition in primates. In human and nonhuman primates, the maternal plasma concentrations of unconjugated E_2_ gradually increase during gestation and reach peak levels at term^1^. Notably, increased levels of circulating 17β-estradiol^2^,^3^ and enhanced estrogen receptor (ER) activity^4^,^5^ are also involved in the proinflammatory cascade, that leads to parturition^6^. The activator protein (AP-1) family of transcription factors regulates various estrogen-induced cellular processes, including inflammation^7^. AP-1 is typically incorporated into c-Jun and c-fos heterodimeric complexes, and the AP-1 levels increase in the myometrium and amnion prior to the onset of labor^8^. These observations indicate that AP-1 plays a key role in the inflammation-mediated induction of labor^9^. COX2 is a highly inducible gene that is expressed at low to undetectable levels in the uterus throughout most of pregnancy, and it is highly up-regulated by proinflammatory cytokines and E_2_ at term^10^,^11^. COX2 catalyzes the production of prostaglandins in the amnion^12^ and plays a crucial physiological role in the initiation of labor by functioning as a potent activator of uterine contractility^13^. PGE2 is upregulated in inflammation-induced preterm delivery, probably because it intensifies the inflammatory response, thereby promoting uterine smooth muscle contraction^14^.

Recent studies have reported that microRNAs (miRNAs) play a role in the regulation of genes that influence uterine quiescence/contractility during pregnancy and labor^15^,^16^,^17^. During the induction of labor, an increase in miR-200 levels inhibits the expression of ZEB1 and ZEB2, thereby facilitating the upregulation of OXTR and GJA1 expression. ZEB1 and ZEB2 decrease the expression of the miR-199a/214 cluster, thereby upregulating COX2 expression and subsequently increasing the synthesis of contractile prostaglandins. The increase in miR-200 expression inhibits another target, STAT5B, thereby facilitating the expression of the gene encoding 20α-HSD and promoting the local metabolism of progesterone (P_4_) in the myometrium. In a more recent study, we have observed an increase in miR-144 expression and a corresponding increase in c-fos and COX2 expression 114 days and 112 days after insemination in female Large White pigs with and without signs of labor onset, respectively^18^. The identification of miRNAs as hormonally regulated modulators of gene expression prompted us to investigate the roles of miRNAs in the synthesis of PGE2 during pregnancy and labor.

In the present study, we report that c-fos, COX2 and miR-144 act as hormonal modulators in the amnion in mice and humans, thereby increasing the expression of miR-144, c-fos and COX2 in the amnion of pregnant mice (15.5 d post-coitum (dpc)) and near term mice (18.5 dpc). E_2_ treatment significantly increased the expression of miR-144, c-fos and COX2 in the mouse amnion and human amniotic cells (WISH). The induction of preterm labor in mice by lipopolysaccharides (LPS) was associated with an increase in miR-144, c-fos, and COX2 expression and the induction of PGE2 expression. miR-144 down-regulated synthesis of PGE2 by direct and indirect repression of COX2, and by direct repression of c-fos, a transcriptional activator of COX2 and miR-144 during pregnancy. E_2_ activated c-fos, thus promoting the expression of miR-144 and COX2; after this increase in COX2, miR-144 partially reduced the expression of COX2, which can be assumed to be a self-protection mechanism against the inflammatory response.

## Results

### miR-144, c-fos and COX2 are up-regulated in pregnant and near term mice

**Figure 1:**
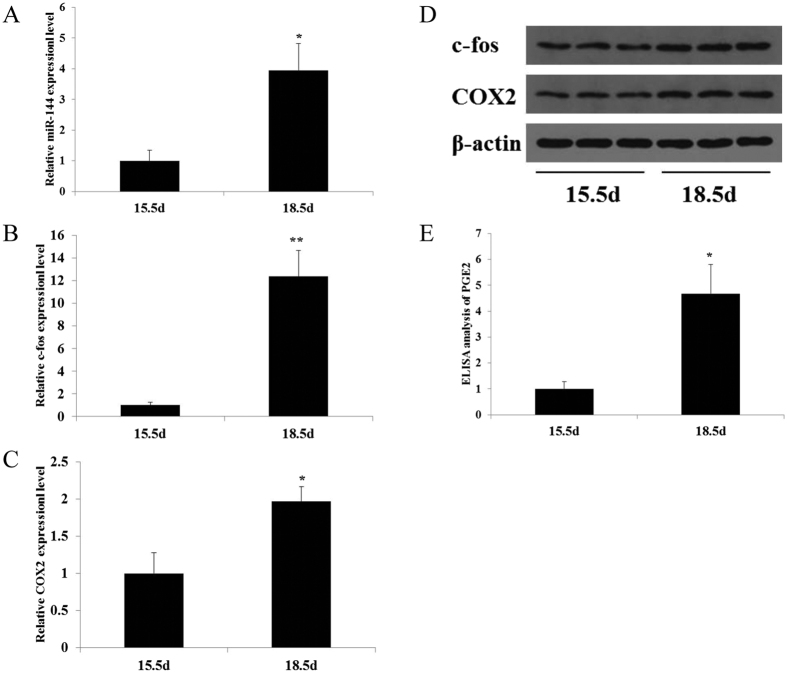
miR-144, c-fos, COX2 and PGE2 are coordinately regulated during pregnancy and near term in the mouse amnion. (**A**) qRT-PCR revealed that miR-144 expression in the mouse amnion significantly increased during the initiation of labor. c-fos and COX2 mRNA (**B,C**) and protein (**E**) levels increased between 15.5 dpc and 18.5 dpc. (**D**) PGE2 levels markedly increased in the amniotic fluid of pregnant mice between 15.5 and 18.5 dpc. The data are presented as the mean ± SEM (*P < 0.05, **P < 0.01; n = 5 mice per group).

In a previous deep sequencing analysis of Large White sows placentas before and at the onset of labor, we found that miR-144, c-fos and COX2 were significantly up-regulated near term^18^. To confirm whether the expression pattern of miR-144, c-fos and COX2 is conserved in mice, qRT-PCR analysis of mouse amniotic tissue at 15.5 and 18.5 dpc was performed, and the results showed that miR-144 (Fig. 1A) as well as c-fos and COX2 (Fig. 1B,C) were increased. Western blot analysis also confirmed the increase in c-fos and COX2 (Fig. 1D). ELISA analysis also indicated an increase in amniotic fluid PGE2 (Fig. 1E). Together, these results indicated that the expression patterns of miR-144, c-fos and COX2 during pregnancy are similar in pigs and mice.

### miR-144, c-fos and COX2 are up-regulated in a mouse models of preterm labor

**Figure 2:**
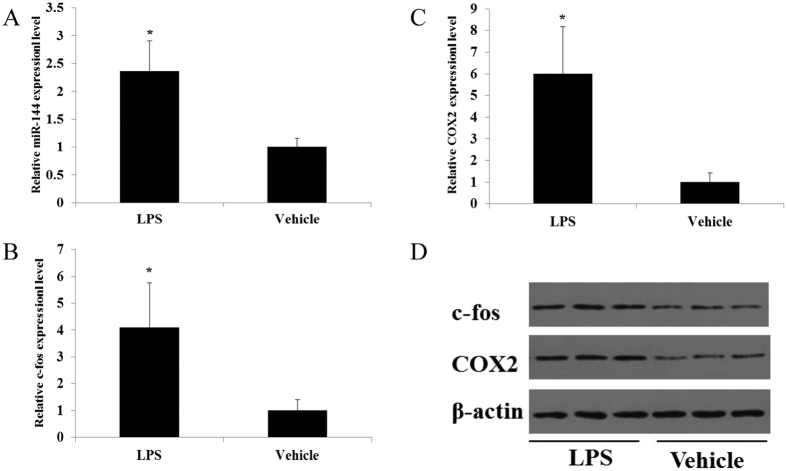
miR-144, c-fos, and COX2 are up-regulated in a mouse model of preterm labor. (**A–D**) Animals received a single s.c. injection of LPS (1.5 μg) into the amniotic sac at 15.5 dpc. Mice were considered to be in labor after the birth of one pup. LPS treatment increased the expression of miR-144 (**A**) c-fos, and COX2 at the mRNA (**B,C**) and protein (**D**) levels. The data are presented as the mean ± SEM. Student’s t-test was used to calculate statistical significance. *P < 0.05, n = 5 mice per treatment group.

To determine whether the changes in the expression of miR-144, c-fos and COX2 were associated with preterm labor, preterm labor was induced by injecting LPS into the amniotic sacs of 15.5 dpc mice^19^. LPS treatment promoted miR-144 expression in the amnion (Fig. 2A) and increased the mRNA and protein levels of c-fos and COX2 (Fig. 2B–D). These findings suggest that up-regulation of the expression of miR-144, c-fos and COX2 in anmion may play a role preterm labor.

### c-fos up-regulates miR-144 and COX2 in WISH cells

**Figure 3:**
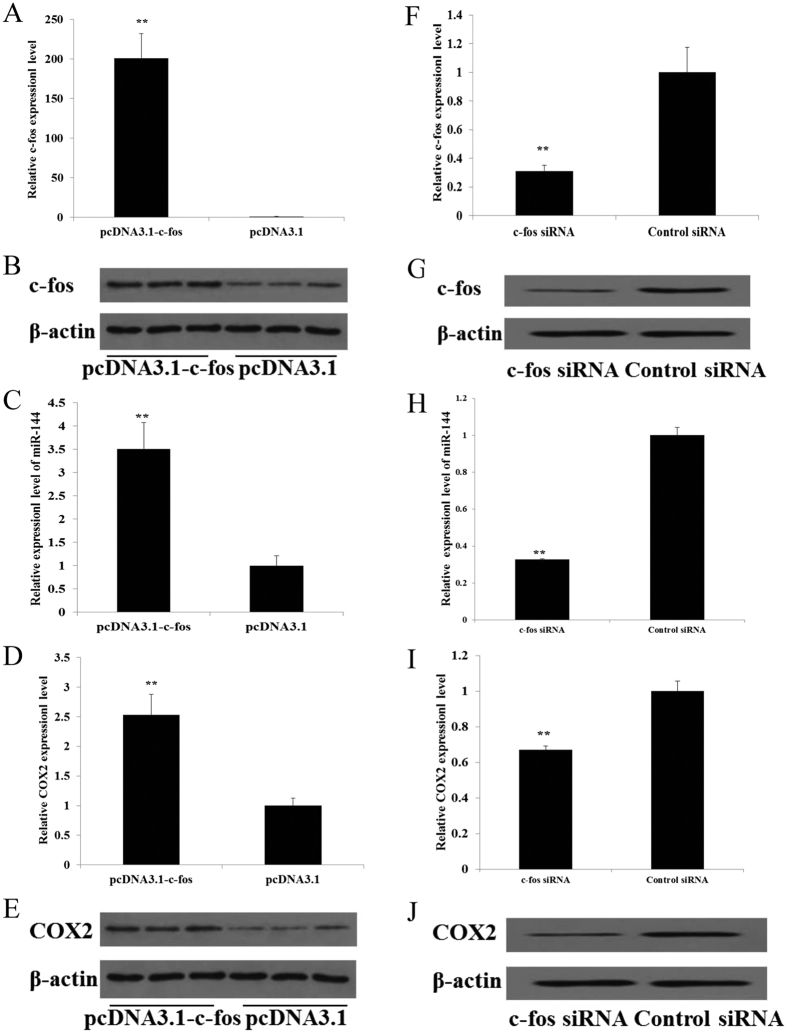
c-fos upregulates miR-144 and COX2 expression in WISH cells. (**A–E**) WISH cells were transfected with the pcDNA3.1-c-fos expression plasmid or an empty vector (control). c-fos mRNA (**A**) and protein (**B**) levels increased 48 h after transfection. The up-regulation of c-fos was associated with a significant increase in miR-144(C) and COX2 mRNA (**D**) and protein (**E**) levels. (**F–J**) WISH cells were transfected with c-fos siRNA or with a scrambled siRNA (control). c-fos mRNA (**F**) and protein (**G**) levels significantly decreased 48 h after transfection. (**H**) The decrease in c-fos expression was associated with a significant increase in miR-144 levels. The down-regulation of c-fos was associated with a significant suppression of COX2 mRNA (**I**) and protein (**J**) levels. The data are presented as the mean ± SD of the results of three independent experiments. Each experiment was conducted in triplicate. *Statistically significant (*P* < 0.05) difference compared with the control.

Recent studies have reported that the c-fos levels increase in the myometrium and placenta during labor^20^,^21^. Although data from previous studies have indicated that c-fos positively regulates COX2 expression^22^, this relationship has not been established in the amnion. Similarly, the association between c-fos and miR-144 has not previously been reported. To investigate whether c-fos regulates miR-144 and COX2 expression in the amnion, a pcDNA3.1 expression plasmid was used for the c-fos transfection of WISH cells to augment c-fos expression at both the mRNA and protein levels (Fig. 3A,B); this transfection led to an increase in the mRNA and protein levels of miR-144 and COX2 (Fig. 3C–E). In contrast, the siRNA-mediated knockdown of c-fos in WISH cells decreased the mRNA and protein levels of miR-144 and COX2 (Fig. 3F–J).

**Figure 4:**
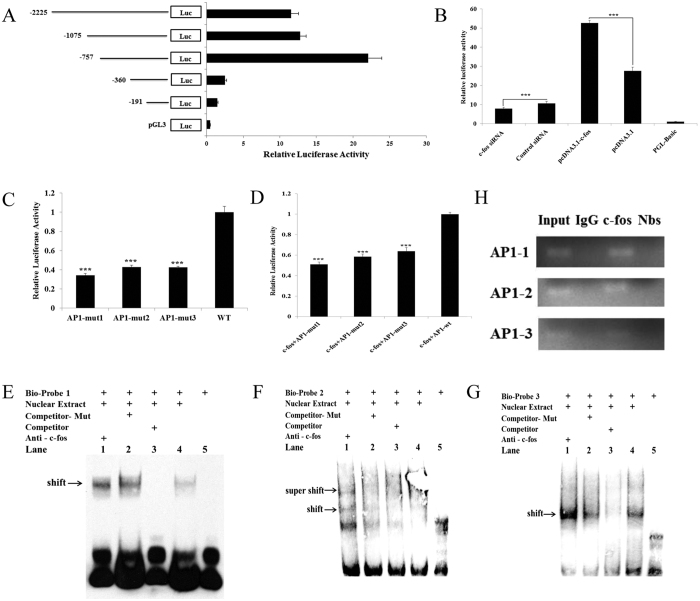
Transcriptional regulation of miR-144. (**A**) Identification of miR-144 promoter activity by using luciferase assays in WISH cells. (**B**) WISH cells were co-transfected with c-fos overexpression vectors and the pGL3-promoter-1075 reporter . The overexpression of c-fos strongly increased luciferase activity. Luciferase activity decreased in WISH cells co-transfected with c-fos siRNA and the pGL3-promoter-1075 reporter. (**C**) A mutation in the AP-1 binding site strongly reduced transcriptional activity compared with wild-type AP-1. (**D**) A strong reduction in transcriptional activity was observed in WISH cells cotransfected with c-fos overexpression vectors and the pGL3-promoter-1075 reporter with an AP-1 binding site mutation compared with the reporter with the wild-type AP-1 sequence. (**E–G**) WISH cells nuclear extracts were incubated with biotin-labelled DNA probes corresponding to the 3 putative AP-1 binding sites in the miR-144 promoter (lanes 2–5). A 200-fold excess of unlabelled DNA probe was included in the reaction to evaluate binding competition. The supershift assay was conducted using an antibody against c-fos (lane 1). (**H**) ChIP assays using antibodies against c-fos were used to evaluate the binding activity of endogenous c-fos to the region of the AP-1 promoter that contains with the putative c-fos response elements. c-fos binding was evaluated using PCR. The data are presented as the mean ± SEM (*P < 0.05).

To further examine whether c-fos regulates miR-144, the miR-144 promoter region was isolated from a luciferase reporter construct, referred to as pGL3-promoter-2225, containing a 2.2-kb region upstream of the precursor miR-144. According to sequence conservation data, four additional luciferase reporter constructs containing decreasing lengths of the upstream sequence (-1075 bp, -757 bp, -360 bp and -191 bp) were derived. The five luciferase reporter constructs and the pGL3-basic plasmid were then transfected separately into WISH cells to determine the basal promoter activity. As shown in Fig. 4A, the pGL3-promoter-757 construct (i.e., the 757-bp putative promoter region) displayed the highest luciferase activity, which was 20-fold higher than that of pGL3-basic. The other two reporter constructs with relatively larger regions of the upstream sequence exhibited a level of luciferase activity similar to that of pGL3-promoter-360, thus suggesting that the transcriptional start site is located in the region between -757 and -360 bp. These results indicated that the promoter activity that triggers miR-144 transcription resides in the region -360 bp upstream of the precursor miR-144. To investigate whether c-fos is involved in miR-144 transcription, the region 1-kb upstream of the precursor miR-144 was analysed using transcription factor binding prediction software. Three putative AP-1 binding sites were identified in the region 1-kb upstream of the miR-144 precursor. Thus, c-fos overexpression vectors and pGL3-promoter-1075 reporter were co-transfected into WISH cells. As shown in Fig. 4B, c-fos overexpression strongly increased luciferase activity. In contrast, luciferase activity decreased in WISH cells co-transfected with c-fos siRNA and the pGL3-promoter-1075 reporter construct (Fig. 4B). The binding site of the AP-1 mutation markedly affected the transcriptional activity compared with that of wild type (Fig. 4C). A similar result was observed in WISH cells overexpressing c-fos (Fig. 4D). We also evaluated the binding properties of AP-1 by using EMSA and supershift assays (Fig. 4E–G). We examined the DNA probes corresponding to the AP-1 binding sites 1, 2 and 3, which contained the putative AP-1 binding sequences and the regions flanking them on both sides in the human miR-144 promoter. Incubating each of the biotin-labelled probes with the WISH cells nuclear extracts caused a major retarded band. Excessive amounts of unlabelled probes prevented each labelled probe from binding with AP-1, as indicated by the dim retarded band. Additionally, the incubation of c-fos antibody caused supershifted bands indicating the presence of c-fos in the binding complex. The *in vitro* recruitment of c-fos to the miR-144 promoter was assessed by using a ChIP assay, which indicated that c-fos was recruited to the miR-144 promoter (Fig. 4H). These results clearly demonstrate that c-fos regulates the transcription of miR-144 and COX2 by binding the potential sites in its promoter.

### miR-144 targets c-fos and COX2

**Figure 5:**
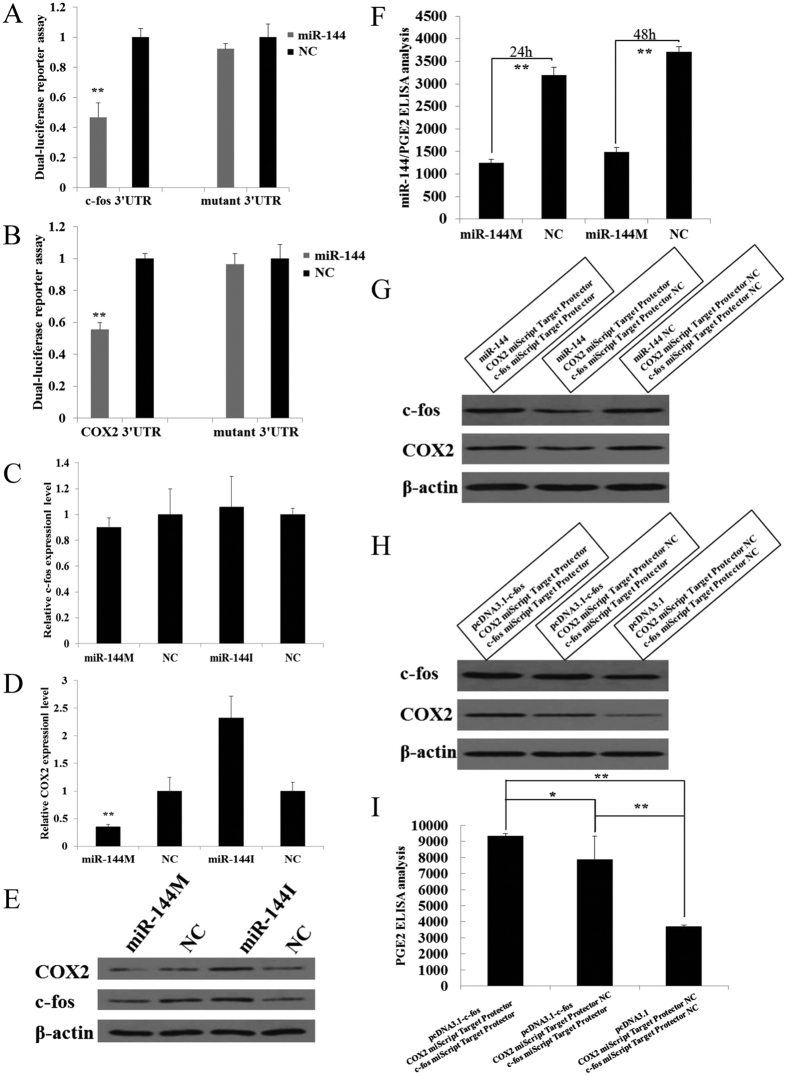
miR-144 directly and indirectly targets c-fos and COX2, and inhibits the synthesis of PGE2. (**A**) Luciferase assays were conducted on HeLa cells cotransfected with either miR-144 or mimics NC and the luciferase:c-fos 3′UTR reporter with the WT or mutant miR-144 binding sites. (**B**) Luciferase assays were conducted on HeLa cells cotransfected with either miR-144 or mimics NC and the luciferase: COX2 3′UTR reporter with the WT or mutant miR-144 binding sites. The luciferase activity in cells cotransfected with miR-144 relative to luciferase activity in cells transfected with the NC are plotted. The mimics NC did not affect reporter activity. The values are presented as the mean ± SD from three independent experiments, each of which was conducted in triplicate. *Statistically significant (*P* < 0.05) difference compared with cells cotransfected with NC. The results of the cotransfection experiments indicate that miR-144 directly targets the c-fos 3′UTR and the COX2 3′UTR. The effect of miR-144 on c-fos and COX2 was abolished when the putative miR-144 binding sites were mutated. (**C–F**) WISH cells were transfected with miR-144 mimics, NC mimics, a miR-144 inhibitor or the NC inhibitor. After 48 h, no significant changes in c-fos mRNA levels were observed (**C**). miR-144 inhibited COX2 mRNA expression (**D**), reduced c-fos and COX2 protein levels (**E**), and decreased PGE2 levels (**F**). (**G**) miR-144 indirectly regulates COX2 via c-fos. COX2 protein levels increased in cells transfected with miR-144 and the c-fos target protector. (**H**) miR-144 partially inhibited COX2 expression. WISH cells were transfected with the c-fos overexpression plasmid and either the COX2 target protector or the COX2 target protector NC. Levels of the COX2 protein and PGE2 increased in cells transfected with the c-fos overexpression plasmid and the COX2 target protector (**I**). The data are presented as the mean ± SD (*P < 0.05).

As mentioned above, the 3′UTR of c-fos and COX2 was found to contain putative binding sites for miR-144. To assess whether miR-144 directly targets c-fos and COX2, we transfected HeLa cells separately with miR-144 mimics and a luciferase reporter plasmid containing a portion of the c-fos 3′UTR or COX2 3′UTR, which resulted in significant down-regulation of the luciferase activity of both the c-fos 3′UTR and COX2 3′UTR. This repression was reversed when the putative miR-144 binding sites were mutated (Fig. 5A,B). Additionally, we investigated whether miR-144 inhibits endogenous c-fos and COX2 expression in human WISH cells. Transfecting the WISH cells with miR-144 mimics significantly decreased the protein level of c-fos and the mRNA and protein levels of COX2 as well as the concentration of PGE2 in the cell supernatants; in contrast, transfecting the WISH cells with miR-144 inhibitors significantly increased the protein level of c-fos and the mRNA and protein levels of COX2 but did not significantly regulate the mRNA level of c-fos (Fig. 5C–F). As shown above, because c-fos can increase the expression of COX2, we hypothesized that miR-144 might indirectly regulate COX2 expression via c-fos. To test this hypothesis, we transfected WHIS cells with either miR-144 mimics/COX2 target protector/c-fos target protector or miR-144 mimics/COX2 target protector/c-fos target protector negative control (NC). After the transduction of miR-144 and c-fos target protector, the protein levels of COX2 increased (Fig. 5G), thus implying that miR-144 can indirectly target COX2 via c-fos. Together, these data indicate that during pregnancy, the expression of c-fos is lower and that miR-144 directly or indirectly targets both c-fos and COX2, ultimately decreasing the PGE2 levels. Because c-fos regulates the transcription of miR-144 and COX2 and because miR-144 targets c-fos and COX2, we hypothesize that with the increase in c-fos and COX2 that occurs in labor, miR-144 might partially inhibit COX2 expression. To verify this hypothesis, we transfected WHIS cells with c-fos overexpression/c-fos target protector/COX2 target protector or c-fos overexpression/c-fos target protector/COX2 target protector NC. After the transduction of the c-fos overexpression plasmid and the COX2 target protector, the protein levels of COX2 increased (Fig. 5H), as did PEG2 synthesis (Fig. 5I), thus indicating that miR-144 can partly inhibit COX2 via the up-regulation of c-fos. c-fos to promote the expression of miR-144 and COX2, but with the increase of COX2, miR-144 partially inhibited the COX2 expression, thereby reducing slightly the secretion of PGE2 , implying that miR-144 reduces PGE2 secretion by section to avoid preterm delivery.

### E_2_ regulates miR-144, c-fos and COX2 in WISH cells and amnion of pregnant mice

**Figure 6:**
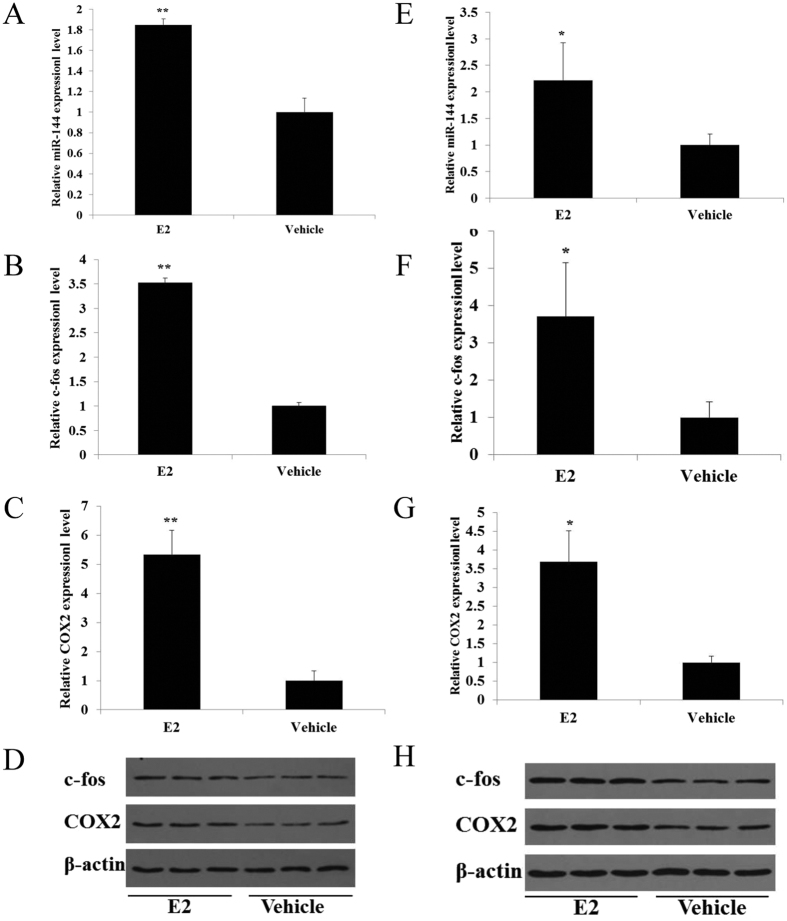
E_2_ regulates miR-144, c-fos and COX2 in the amnion of pregnant mice and in WISH cells. (**A–D**) WISH cells were treated with 10 nM E_2_ and harvested 24 h later. E_2_ treatment increased miR-144 (**A**) c-fos, and COX2 mRNA (**B,C**) and protein (**D**) levels. (**E–H**) Animals received a single s.c. injection of E_2_ (1 μg) into the amniotic sac at 15.5 dpc. Amniotic tissues were harvested 24 h later. E_2_ treatment increased miR-144 (**E**) c-fos, and COX2 mRNA (**F,G**) and protein (**H**) levels. The data are presented as the mean ± SD values. Student’s t-test was used to calculate statistical significance (*P < 0.05).

E_2_ promotes an inflammatory response in the uterus and antagonizes the anti-inflammatory actions of P_4_/PR^4^,^23^. Moreover, the circulating E_2_ levels increase markedly near term in a number of species^2^,^3^,^24^. Treating rats and cultured fibroblasts derived from human uterine endometrium with E_2_ specifically induces c-fos, which is an inflammation-related transcription factor^7^,^25^,^26^. E_2_ has also been reported to promote COX2 expression in the myocardium^27^. To assess the effects of E_2_ on the expression of miR-144, c-fos and COX2 in the amnion, WISH cells were treated with 10 nM E_2_, and mice were administered an amniotic sac injection of E_2_. E_2_ injection, which led to a moderate increase in the expression of miR-144, c-fos and COX2 in both the amnion and WISH cells (Fig. 6). Collectively, these findings suggest that E_2_ promotes the expression of miR-144, c-fos and COX2 and that miR-144 and COX2 are probably mediated, in part, via the induction of c-fos by E_2_.

## Discussion

Understanding of the mechanisms underlying the onset of parturition has increased considerably in recent years^28^. It is clear that E_2_, acting through ER, is essential for maintaining myometrial quiescence and that an enhanced inflammatory response caused by signals from both the mother and foetus promotes the progression to labor^28^. The amnion plays a crucial role in human parturition, and all of the findings in the present study indicate that the post-transcriptional regulation of amniotic gene expression by miRNAs is an integral component of human parturition^16^,^17^,^29^.

Despite growing insights into the signals and pathways leading to the initiation of labor, much remains to be discovered regarding the mechanisms by which the myometrium is transformed from a refractory near-quiescent state to a highly contractile unit capable of responding to a variety of signals from the amnion^16^,^17^,^29^. By elucidating a unique regulatory pathway involving the miR-44 and its targets, c-fos and COX2, our research provides some insights into the mechanisms though which E_2_ modulates the synthesis of PGE2 in the amnion during pregnancy and labor.

Notably, increased levels of circulating E_2_^2^,^3^ and enhanced ER activity^4^,^5^ are involved in the proinflammatory cascade that promotes parturition. Fetal membranes can be synthetic free E_2_^30^. Romero has reported that spontaneous human parturition at term is associated with a significant increase in the concentration of E_2_ in the amniotic fluid^31^. When the E_2_ levels in the local tissue of the uterus near a particular threshold, the E_2_/P_4_ balance is disrupted, and labor becomes inevitable. E_2_ activation appears to be a prime player in the initiation of labor because the phenotypic changes observed in the amnion are induced by E_2_ injection. E_2_ can induce the expression of c-fos, which is increased in the myometrium and amnion prior to the onset of labor^32^. Recently, Liu *et al.* have demonstrated the functional significance of miR-144 and the regulation feedback loop of c-fos in the migration and invasion of hepatoma cells^33^. Similar results were obtained in our research. COX2 is a key enzyme in the synthesis of prostaglandins, and there is a good correlation between the COX2 and PGE2 expression levels in the amnion^34^. The regulation of COX2 is a complex process that involves both transcriptional and post-transcriptional events^35^. It has been shown that c-fos can increase the expression of COX2 in both human amniotic cells and the myometrium^36^,^37^.

Our findings indicate that elevated circulating E_2_ levels throughout most of pregnancy directly up-regulate the expression of c-fos in the amnion. After 17.5 dpc, with an increase in the E_2_ levels^21^, the mRNA and protein levels of c-fos in the amnions of pregnant mice are significantly increased. Because c-fos directly binds and promotes the miR-144 promoter, an increase in c-fos results in a reciprocal up-regulation of miR-144^4^,^23^ (Fig. 3E). We also found that c-fos up-regulates COX2 expression. From these collective findings, we conclude that the increase in miR-144 and COX2 that occurs during late gestation in response to LPS treatment is attributable to the rise of c-fos expression but that the decrease in miR-144 and COX2 that occurs before late gestation is attributable to the down-regulation of c-fos expression. Further, the expression relationship among miR-144, c-fos and COX2 after E_2_ addition at different time points could be investigated in the subsequent experiment.

In addition to characterizing the regulation of c-fos, we explored the functional role of miR-144 and COX2 in the amnion in near term mice. Recent studies have indicated that during pregnancy and labour, the myometrium, cervix, and foetal membranes are affected by several miRNAs, including miR-200, miR-199a-3p, miR-214, miR-34b, miR-34c, miR-338 and miR-223, which suggests the key collaborative roles of miRNAs in the hormonal control of myometrial quiescence and contractility during pregnancy and labor^15^,^16^,^17^,^38^,^39^,^40^. Chakrabarty *et al.* have recently demonstrated the functional significance of amnion expression of miR-143 and its regulation of COX2 in labor at term^29^. In the present study, the overexpression analysis of miR-144 mimics in cultured human WISH cells indicated that miR-144 downregulates the expression of c-fos and COX2 and that c-fos interference inhibits the synthesis of PGE2 in the amnion during pregnancy and labor. Further tests indicated that miR-144 indirectly targets COX2 via c-fos.

The up-regulation of COX2 expression in the amnion may increase the PGE2 concentration, thus leading to the contraction of myometrial strips, which may be the cause of labor onset^12^. PGE2 is upregulated in inflammatory-induced preterm delivery, probably due to the intensification of the inflammatory response, which, in turn, causes uterine smooth muscle contraction^41^. COX2 inhibitors can decrease both COX2 expression and PGE2 concentration, thereby inhibiting the contraction of myometrial strips and delaying labor onset^14^. In the present study, the elevated circulating E_2_ levels directly up-regulate the expression of c-fos in the amnion throughout most of pregnancy. With the increased expression of c-fos, the expression of COX2 is also upregulated, but miR-144 partially inhibits COX2 expression, thus slightly reducing the PGE2 secretion. We hypothesize that miR-144 reduces PGE2 secretion by section to avoid the onset of premature delivery. Therefore, the labor-control mechanism is a multi-level complex network rather than an isolated single-level one, though the details of the network remain to be further elucidated.

**Figure 7:**
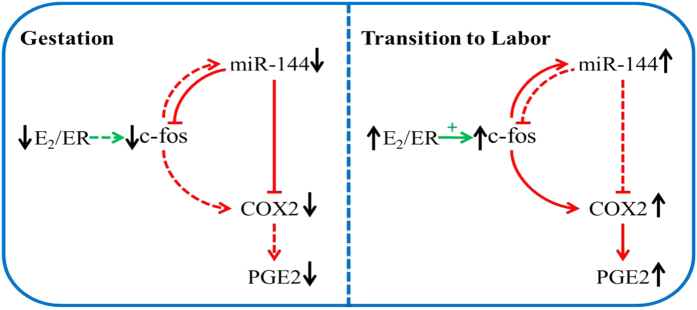
Schematic diagram of the regulation of E_2_ during pregnancy and labor via the miR-144/c-fos/COX2/PGE2 axis.

In summary, c-fos is a key E_2_ target gene in amnion that promotes the synthesis of PGE2, and it is associated with COX2 and miR-144. The decrease in E_2_ levels before term leads to down-regulation of c-fos gene expression, which in turn results in the down-regulation of miR-144 and COX2 expression, and miR-144 directly and indirectly targets c-fos and COX2, all of which reduce the secretion of PGE2 throughout most of pregnancy (Fig. 7). Near term, signals from both the foetus and mother cause an increased inflammatory response that leads to an increase in the local E_2_ levels. This increase up-regulates the expression of the c-fos gene, which leads to the increased expression of miR-144 and COX2. Then, the resulting elevated miRNAs partly repress COX2, thus resulting in a slight down-regulation of PGE2 secretion and suppression of PGE2 overexpression, and leading to the final inhibition of premature delivery (Fig. 7). Overall, our findings implicate a previously undiscovered pathway in the regulation of PGE2 synthesis in the amnion during pregnancy and parturition and may open an avenue for developing effective therapeutics to prevent preterm labor.

## Materials and Methods

### Mouse tissue preparation

All of the studies involving animals were conducted according to the regulation (No. 5 proclamation of the Standing Committee of Hubei People’s Congress) approved by the Standing Committee of Hubei People’s Congress, P. R. China. The sample collection procedure used in this study was approved by the Ethics Committee of Huazhong Agricultural University (permit number No. 30700571). The animals were allowed ad libitum access to food and water. They were housed under standard conditions and were humanely sacrificed to minimize suffering, in accordance with the approved guidelines. Amnion tissues were isolated at 15.5–18.5 dpc after the delivery of the first pup (in labor) as previously described^17^.

### E_2_ treatment studies

WISH cells were plated in 6-well dishes. When the cells reached approximately 70% confluence, they were cultured in serum-free medium containing the vehicle controls or 100 nM E_2_ for 6 h. Pregnant 15.5 dpc Kunming mice were injected with E_2_ (Sigma) (1 μg in 0.3 ml of the sesame oil vehicle) or the vehicle alone. The amnions were harvested 24 h after the injection and flash frozen for subsequent protein, mRNA, and miRNA analyses.

### Mouse model of preterm labor by LPS-induced inflammation

The induction of preterm labor using LPS was conducted as previously described^17^. LPS (1.5 μg in 50 μL of PBS) or sterile PBS (vehicle) was injected into the amniotic sac of each mouse, and the uterus was carefully reinserted into the abdominal cavity. The abdominal muscle wall and skin were subsequently sutured, and the mouse was allowed to recuperate. There was a high rate of preterm labor in the LPS-injected mice, and no instances of preterm labor were observed in the vehicle-injected mice. The LPS-injected mice were sacrificed after the birth of 1 pup, and the gestation-matched controls were sacrificed immediately afterwards.

### Cell cultures

WISH cells were cultured in MEM medium (HyClone), and HeLa cells were cultured in DMEM (HyClone) supplemented with 10% foetal bovine serum (Gibco) and streptomycin. The cells were incubated at 37 °C with 5% CO_2_.

### Transfection

To evaluate the effects of the c-fos transcription factor, c-fos was amplified from human cDNA and cloned into the pcDNA3.1 vector. WISH cells were transfected with 100 ng of the pcDNA3.1 plasmid expressing c-fos or the empty pcDNA3.1 plasmid and harvested 48 h after the transfection. To evaluate the effects of c-fos knockdown, WISH cells were transfected with either 20 nM control small interfering RNA (siRNA) or 20 nM c-fos siRNA and harvested 48 h after the transfection. The sequence of siRNA duplexes for c-fos and negative control (scrambled) was as follows: c-fos siRNA targeting 5′- GCAAGGUGGAACAGUUAUC-3′.

For the miRNA mimic studies, the cells were transfected with 20 nM scrambled control mimic, the miR-144 mimic or the miR-144 inhibitor (Ribobio, Guangzhou, China) using Lipofectamine RNAiMAX Transfection Reagent (Invitrogen). The cells were harvested for the mRNA and protein analyses 48 h after the transfection.

For the miRNA target protector studies, the cells were transfected with 1000 nM c-fos miScript Target Protector (QIAGEN) and 20 nM miR-144 mimic using Lipofectamine RNAiMAX Transfection Reagent (Invitrogen). Next, the cells were transfected with 1000 nM COX2 miScript Target Protector (QIAGEN) and 1 μg of the c-fos overexpression vector using Lipofectamine 3000 Transfection Reagent (Invitrogen). The cells were harvested for mRNA and protein analyses 48 h after transfection. MiScript Target Protectors are single-stranded, modified RNAs that interfere with the interaction of a specific miRNA with a single target and do not influence the interaction of an miRNA with its other targets. The sequences of the c-fos and COX2 miScript Target Protectors were 5′-AAATAGCTATATCCATGTACTGTAGTTTTT-3′ and 5′-GTCACTGACATTTAATGGTACTGTATATTA-3′, respectively.

### Luciferase reporter assays

The TargetScan prediction software (http://www.targetscan.org) was used to identify the putative miR-144 binding sites in the 3′UTRs of mouse and human c-fos and COX2. To construct the reporter plasmid expressing pmirGLO-c-fos 3′UTR or the COX2 3′UTR, the c-fos 3′UTR and COX2 3′UTR regions were amplified from human cDNA, and the PCR product was digested with MssI and NheI. The fragment, which spans 425 bp of the c-fos 3′UTR and 499 bp of the COX2 3′UTR, was cloned into the pmirGLO Dual Luciferase miRNA Target Expression Vector (Promega). For the analysis of the 3′UTR mutations, four nucleotides in the putative miR-144 binding site were mutated (c-fos 3′UTR CCATGTACTGT-5′ to 3′-CCATGTAAGTG-5′ and COX2 3′UTR 3′-CATTTAATGGTACTGTA-5′ to 3′-CATTTAATGGTACGAGC-5′). HeLa cells were co-transfected with the reporter plasmids and 20 nM miR-144 or the control, and relative luciferase activity was assayed 24 h later.

To construct the pGL3-promoter-2225, promoter-1075, promoter-757, promoter-360 and promoter-191 luciferase reporter plasmids, each region of the miR-144 promoter was amplified from human genomic DNA and cloned into the pGL3-basic plasmid (Promega). For the luciferase assay, WISH cells were plated in 24-well plates and co-transfected 24 h later with 0.2 μg of the pGL3 reporter plasmid, 0.002 μg of the Renilla plasmid (Promega) and 50 nM miR-144 mimic.

The miR-144 promoter reporter plasmids with specific single mutations in the miR-144 binding site of the AP-1 transcription factor AP-1 binding site were generated by using fusion PCR. HeLa cells were co-transfected with the pGL3-AP1-mut reporter plasmid and a recombinant plasmid expressing pcDNA3.1-c-fos or an empty vector. Relative luciferase activity was assayed 24 h after the transfection of the luciferase reporter.

### Quantitative RT-PCR

For gene expression analysis, total RNA was reverse transcribed using a PrimeScript RT reagent kit (TaKaRa). The quantitative RT-PCR analysis was conducted using SYBR Green Real-time PCR Master Mix (TOYOBO) and a CFX96 Real-time System (Bio-Rad). The sequences of the primers used for quantitative RT-PCR were as follows: mouse c-fos forward: 5′-AGGTCGGTGTGAACGGATTTG-3′, mouse c-fos reverse: 5′′-TGTAGACCATGTAGTTGAGGTCA-3′, mouse COX-2 forward: 5′-CAGCCAGGCAGCAAATCC-3′, mouse COX-2 reverse: 5′-ACATTCCCCACGGTTTTGAC-3′, human c-fos forward: 5′-TACTACCACTCACCCGCAGAC-3′, human c-fos reverse: 5′- GAATGAAGTTGGCACTGGAGA-3′, human COX-2 forward: 5′-TTCCAGATCCAGAGCTCATTAAA-3′, human COX-2 reverse: 5′-CCGGAGCGG GAAGAACT-3′, miR-144 forward: 5′-TCGGCAGGTACAGTATAGATGAT-3′, and miR-144 reverse: 5′-TCAACTGGTGTCGTGGAGTCGGC-3′. U6 RNA and β-actin were used as endogenous internal controls, and the relative expression levels were calculated with the 2^−ΔΔCT^ method.

### Immunoblotting

Cytoplasmic and nuclear protein extracts were isolated from cellular and tissue samples using RIPA buffer (Beyotime) supplemented with a protease inhibitor cocktail and 1 mM PMSF (Beyotime). The expression levels of the proteins of interest were analysed separately using primary polyclonal antibodies against COX-2 and c-fos (Boster) at a dilution of 1:200. The horseradish peroxidase-conjugated goat anti-rabbit secondary antibody (Boster) was used at a dilution of 1:5000. The blots were stripped and re-probed with antibodies against β-actin (Abcam), which was used as a loading control.

### Electrophoretic mobility shift assay (EMSA)

Nuclear proteins from WISH cells were extracted by using a Nucleoprotein Extraction Kit (Sangon). Synthetic complementary oligonucleotides containing the AP-1-binding site of the miR-144 promoter were 3′-biotinylated and annealed. The wild-type AP-1-1 oligonucleotide sequence is 5′- GCTGGGAATAGAAATTTAGGCTGACGGGTAGGGTGAGCTCGGCTGCCA-3′, and the mutant sequence is 5′- GCTGGGAATAGAAATTTAGGCAAGAAATGCGGGTGAGCTCGGCTGCCA-3′. The wild-type AP-1-2 oligonucleotide sequence is 5′- CTTCTAGGGAAAGGGGCCAGTGACCCTTGTCATGGACTCTAGCAGGGC-3′, and the mutant sequence is 5′- CTTCTAGGGAAAGGGGCCAGTGAAAACGCTCATGGACTCTAGCAGGGC-3′. The wild-type AP-1-3 oligonucleotide sequence is 5′- CCATAACCCACCTGGGCTGTGCCTGACCACAGAATCAAGGAGACGCTG-3′, and the mutant sequence is 5′- CCATAACCCACCTGGGCTGTGCCGCCAGGCAGAATCAAGGAGACGCTG-3′. The DNA-protein-binding assays were conducted using horseradish peroxidase-conjugated streptavidin and the LightShift Chemiluminescent EMSA Kit (Thermo) according to the manufacturer’s instructions.

### CHIP

ChIP experiments were conducted to assess the binding of endogenous c-fos to the miR-144 promoter in WISH cells using a ChIP Assay Kit (Millipore). Precleared chromatin was incubated with the c-fos antibody (Abcam) or the nonimmune IgG control overnight at 4 °C. The immunocomplexes were isolated on Protein A agarose beads with a ChIP Assay Kit (catalogue no. 17–295; Millipore). The chromatin complexes were eluted from the beads, and the DNA cross-linking was subsequently reversed. Purified DNA from the samples and the input controls was analysed for the presence of miR-144 promoter sequences containing putative c-fos response elements using qPCR and the following primers: AP-1-1 (forward: 5′-ATTTAGGCTGACGGGTAG-3′; reverse: 5′- AGTGGTGGTAGGCAATGT-3′), AP-1-2 (forward: 5′- TGGAGATGGGAGTGAAGG-3′; reverse: 5′- CCTGGGTGGGGCACTTTT-3′), and AP-1-3 (forward: 5′- CCTGGGTCCCTATGAGAT-3′; reverse: 5′- GGCAGAACAGGACAGGTC-3′). We also synthesized primers targeting the miR-144 promoter sequence that did not contain the c-fos binding site (Nbs).

### ELISA

The levels of PGE2 in WISH cells supernatants were determined using enzyme-linked immunosorbent assay (ELISA). The medium in which the WISH cells were cultured was collected and centrifuged at 13,000 g for 15 min to pellet the debris. The ELISAs were conducted according to the manufacturer’s instructions (QIAGEN). The colorimetric reaction was measured at 450 nm.

### Statistical Analysis

Excel (Microsoft) was used to analyse the data. Statistical significance was determined using the two-tailed Student’s t test and *P* < 0.05 was considered to be statistically significant.

## Additional Information

**How to cite this article**: Li, H. *et al.* miR-144 and targets, c-fos and cyclooxygenase-2 (COX2), modulate synthesis of PGE2 in the amnion during pregnancy and labor. *Sci. Rep.* **6**, 27914; doi: 10.1038/srep27914 (2016).

## References

[b1] Albrecht E. D. & Pepe G. J. Placental steroid hormone biosynthesis in primate pregnancy. Endocr. Rev. 11, 124–150, doi: 10.1210/edrv-11-1-124 (1990). 2180685

[b2] Buster J. E. *et al.* Interrelationships of circulating maternal steroid concentrations in third trimester pregnancies. II. C18 and C19 steroids: estradiol, estriol, dehydroepiandrosterone, dehydroepiandrosterone sulfate, delta 5-androstenediol, delta 4-androstenedione, testosterone, and dihydrotestosterone. J. Clin. Endocr. Metab. 48, 139–142, doi: 10.1210/jcem-48-1-139 (1979). 154525

[b3] Challis J. R. Sharp increase in free circulating estrogens immediately before parturition in sheep. Nature 229, 208 (1971). 4923273 10.1038/229208a0

[b4] Mesiano S. *et al.* Progesterone withdrawal and estrogen activation in human parturition are coordinated by progesterone receptor A expression in the myometrium. J. Clin. Endocr. Metab. 87, 2924–2930, doi: 10.1210/jcem.87.6.8609 (2002). 12050275

[b5] Wu W. X., Myers D. A. & Nathanielsz P. W. Changes in estrogen receptor messenger ribonucleic acid in sheep fetal and maternal tissues during late gestation and labor. Am. J. Obst. Gynecol. 172, 844–850 (1995). 7892873 10.1016/0002-9378(95)90009-8

[b6] Bollapragada S. *et al.* Term labor is associated with a core inflammatory response in human fetal membranes, myometrium, and cervix. Am. J. Obst. Gynecol. 200, 104.e101–111, doi: 10.1016/j.ajog.2008.08.032 (2009). 19121663

[b7] Fujimoto J., Ilori M., Ichigo S., Morishita S. & Tamaya T. Estrogen induces the expression of c-fos and c-jun genes in fibroblasts derived from human uterine endometrium. Exp. Clin. Endocr. Diab. 103, 378–385, doi: 10.1055/s-0029-1211382 (1995). 8788311

[b8] Mitchell J. A. & Lye S. J. Differential expression of activator protein-1 transcription factors in pregnant rat myometrium. Bio. Reprod. 67, 240–246 (2002). 12080023 10.1095/biolreprod67.1.240

[b9] MacIntyre D. A. *et al.* Activator protein 1 is a key terminal mediator of inflammation-induced preterm labor in mice. Faseb. J. 28, 2358–2368, doi: 10.1096/fj.13-247783 (2014). 24497579

[b10] Engstrom T. The regulation by ovarian steroids of prostaglandin synthesis and prostaglandin-induced contractility in non-pregnant rat myometrium. Modulating effects of isoproterenol. J. Endocrinol. 169, 33–41 (2001). 11250644 10.1677/joe.0.1690033

[b11] Tsuboi K. *et al.* Uterine expression of prostaglandin H2 synthase in late pregnancy and during parturition in prostaglandin F receptor-deficient mice. Endocrinology 141, 315–324, doi: 10.1210/endo.141.1.7236 (2000). 10614653

[b12] Weaver-Mikaere L., Gunn A. J., Mitchell M. D., Bennet L. & Fraser M. LPS and TNF alpha modulate AMPA/NMDA receptor subunit expression and induce PGE2 and glutamate release in preterm fetal ovine mixed glial cultures. J. Neuroinflamm. 10, 153, doi: 10.1186/1742-2094-10-153 (2013). PMC3878505 24344780

[b13] Gibb W. The role of prostaglandins in human parturition. Ann. Medicine 30, 235–241 (1998). 10.3109/07853899809005850 9677008

[b14] Rac V. E. *et al.* Dose-dependent effects of meloxicam administration on cyclooxygenase-1 and cyclooxygenase-2 protein expression in intrauterine tissues and fetal tissues of a sheep model of preterm labor. Reprod. Sci. 14, 750–764, doi: 10.1177/1933719107309042 (2007). 18089593

[b15] Williams K. C., Renthal N. E., Gerard R. D. & Mendelson C. R. The microRNA (miR)-199a/214 cluster mediates opposing effects of progesterone and estrogen on uterine contractility during pregnancy and labor. Mol. Endocrinol. 26, 1857–1867, doi: 10.1210/me.2012-1199 (2012). 22973051 PMC3487626

[b16] Williams K. C., Renthal N. E., Condon J. C., Gerard R. D. & Mendelson C. R. MicroRNA-200a serves a key role in the decline of progesterone receptor function leading to term and preterm labor. P. Natl. Acad. Sci. USA. 109, 7529–7534, doi: 10.1073/pnas.1200650109 (2012). PMC3358858 22529366

[b17] Renthal N. E. *et al.* miR-200 family and targets, ZEB1 and ZEB2, modulate uterine quiescence and contractility during pregnancy and labor. P. Natl. Acad. Sci. USA. 107, 20828–20833, doi: 10.1073/pnas.1008301107 (2010). PMC2996411 21079000

[b18] Li H. *et al.* Integrated analysis of miRNA/mRNA network in placenta identifies key factors associated with labor onset of Large White and Qingping sows. Sci. Rep. 5, 13074, doi: 10.1038/srep13074 (2015). 26272496 PMC4536519

[b19] Bariani M. V. *et al.* Role of the endocannabinoid system in the mechanisms involved in the LPS-induced preterm labor. Reproduction 150, 463–472, doi: 10.1530/rep-15-0211 (2015). 26347521

[b20] Chan Y. W., van den Berg H. A., Moore J. D., Quenby S. & Blanks A. M. Assessment of myometrial transcriptome changes associated with spontaneous human labor by high-throughput RNA-seq. Exp. Physiol. 99, 510–524, doi: 10.1113/expphysiol.2013.072868 (2014). 24273302

[b21] Cindrova-Davies T. *et al.* Oxidative stress, gene expression, and protein changes induced in the human placenta during labor. Am. J. pathol. 171, 1168–1179, doi: 10.2353/ajpath.2007.070528 (2007). 17823277 PMC1988867

[b22] Wu M. H. *et al.* Endothelin-1 enhances cell migration through COX-2 up-regulation in human chondrosarcoma. Bba. Gen. Subjects 1830, 3355–3364, doi: 10.1016/j.bbagen.2013.03.014 (2013). 23523690

[b23] Tibbetts T. A., Conneely O. M. & O’Malley B. W. Progesterone via its receptor antagonizes the pro-inflammatory activity of estrogen in the mouse uterus. Biol. Reprod. 60, 1158–1165 (1999). 10208978 10.1095/biolreprod60.5.1158

[b24] Kamel R. M. The onset of human parturition. Arch. Gynecol. Obstet. 281, 975–982, doi: 10.1007/s00404-010-1365-9 (2010). 20127346

[b25] Weisz A. & Bresciani F. Estrogen induces expression of c-fos and c-myc protooncogenes in rat uterus. Mol. Endocrinol. 2, 816–824, doi: 10.1210/mend-2-9-816 (1988). 3173352

[b26] Zeidan M. A. *et al.* Estradiol modulates medial prefrontal cortex and amygdala activity during fear extinction in women and female rats. Bio. Psychiat. 70, 920–927, doi: 10.1016/j.biopsych.2011.05.016 (2011). 21762880 PMC3197763

[b27] Booth E. A., Flint R. R., Lucas K. L., Knittel A. K. & Lucchesi B. R. Estrogen protects the heart from ischemia-reperfusion injury via COX-2-derived PGI2. J. Cardiovasc. Pharm. 52, 228–235, doi: 10.1097/FJC.0b013e3181824d59 (2008). 18806603

[b28] Mendelson C. R. Minireview: fetal-maternal hormonal signaling in pregnancy and labor. Mol. Endocrinol. 23, 947–954, doi: 10.1210/me.2009-0016 (2009). 19282364 PMC2703595

[b29] Kim S. Y. *et al.* miR-143 regulation of prostaglandin-endoperoxidase synthase 2 in the amnion: implications for human parturition at term. PloS one 6, e24131, doi: 10.1371/journal.pone.0024131 (2011). 21915288 PMC3168490

[b30] Mitchell B. F., Cross J., Hobkirk R. & Challis J. R. Formation of unconjugated estrogens from estrone sulfate by dispersed cells from human fetal membranes and decidua. J. Clin. Endocr. Metab. 58, 845–849, doi: 10.1210/jcem-58-5-845 (1984). 6707190

[b31] Romero R., Scoccia B., Mazor M., Wu Y. K. & Benveniste R. Evidence for a local change in the progesterone/estrogen ratio in human parturition at term. Am. J. Obstet. Gynecol. 159, 657–660 (1988). 2971319 10.1016/s0002-9378(88)80029-2

[b32] Lim R. & Lappas M. Differential expression of AP-1 proteins in human myometrium after spontaneous term labor onset. Eur. J. Obst. Gyn. B. 177, 100–105, doi: 10.1016/j.ejogrb.2014.04.016 (2014). 24784710

[b33] Liu J. J. *et al.* A novel AP-1/miR-101 regulatory feedback loop and its implication in the migration and invasion of hepatoma cells. Nucleic Acids Res. 42, 12041–12051, doi: 10.1093/nar/gku872 (2014). 25260594 PMC4231742

[b34] Lee D. C. *et al.* Evidence for a spatial and temporal regulation of prostaglandin-endoperoxide synthase 2 expression in human amnion in term and preterm parturition. J. Clin. Endocr. Metab. 95, E86–91, doi: 10.1210/jc.2010-0203 (2010). 20519349 PMC2936056

[b35] Harper K. A. & Tyson-Capper A. J. Complexity of COX-2 gene regulation. Biochem. Soc. T 36, 543–545, doi: 10.1042/bst0360543 (2008). 18482003

[b36] Mohan A. R., Sooranna S. R., Lindstrom T. M., Johnson M. R. & Bennett P. R. The effect of mechanical stretch on cyclooxygenase type 2 expression and activator protein-1 and nuclear factor-kappaB activity in human amnion cells. Endocrinology 148, 1850–1857, doi: 10.1210/en.2006-1289 (2007). 17218407

[b37] Loudon J. A., Sooranna S. R., Bennett P. R. & Johnson M. R. Mechanical stretch of human uterine smooth muscle cells increases IL-8 mRNA expression and peptide synthesis. Mol. Hum. Reprod. 10, 895–899, doi: 10.1093/molehr/gah112 (2004). 15489245

[b38] Hassan S. S. *et al.* MicroRNA expression profiling of the human uterine cervix after term labor and delivery. Am. J. Obstet. Gynecol. 202, 80.e81–88, doi: 10.1016/j.ajog.2009.08.016 (2010). 19889381

[b39] Montenegro D. *et al.* Differential expression of microRNAs with progression of gestation and inflammation in the human chorioamniotic membranes. Am. J. Obstet. Gynecol. 197, 289.e281–286, doi: 10.1016/j.ajog.2007.06.027 (2007). 17826424 PMC2810125

[b40] Montenegro D. *et al.* Expression patterns of microRNAs in the chorioamniotic membranes: a role for microRNAs in human pregnancy and parturition. J. Pathol. 217, 113–121, doi: 10.1002/path.2463 (2009). 18991333 PMC4160233

[b41] Musilova I. *et al.* Amniotic fluid prostaglandin E2 in pregnancies complicated by preterm prelabor rupture of the membranes. J. Matern. Fetal. Neonatal. Med. 23, 1–9, doi: 10.3109/14767058.2015.1112372 (2015). 26512976

